# Robust Time-of-Arrival Location Estimation Algorithms for Wildlife Tracking

**DOI:** 10.3390/s23239460

**Published:** 2023-11-28

**Authors:** Eitam Arnon, Shlomo Cain, Assaf Uzan, Ran Nathan, Orr Spiegel, Sivan Toledo

**Affiliations:** 1 School of Zoology, Tel Aviv University, Tel Aviv 69978, Israel; 2 The Alexander Silberman Institute of Life Science, The Hebrew University of Jerusalem, Jerusalem 91904, Israel; 3 The Blavatnik School of Computer Science, Tel Aviv University, Tel Aviv 69978, Israel

**Keywords:** location estimation, robust estimation, outlier removal, time-of-arrival localization, wildlife tracking

## Abstract

Time-of-arrival transmitter localization systems, which use measurements from an array of sensors to estimate the location of a radio or acoustic emitter, are now widely used for tracking wildlife. Outlier measurements can severely corrupt estimated locations. This article describes a new suite of location estimation algorithms for such systems. The new algorithms detect and discard outlier time-of-arrival observations, which can be caused by non-line-of-sight propagation, radio interference, clock glitches, or an overestimation of the signal-to-noise ratio. The new algorithms also detect cases in which two locations are equally consistent with measurements and can usually select the correct one. The new algorithms can also infer approximate altitude information from a digital elevation map to improve location estimates close to one of the sensors. Finally, the new algorithms approximate the covariance matrix of location estimates in a simpler and more reliable way than the baseline algorithm. Extensive testing on real-world data involving mobile transmitters attached to wild animals demonstrates the efficacy of the new algorithms. Performance testing also shows that the new algorithms are fast and that they can easily cope with high-throughput real-time loads.

## 1. Introduction

Tracking the movement of many species of wild animals remains highly challenging. For many species, the only viable high-throughput high-resolution tracking technology is time-of-arrival (ToA) transmitter localization [1]. Similar techniques are now also used to track livestock [2]. In such systems, a network of radio or acoustic sensors (receivers) estimate the times of arrival of a signal emitted by a transmitter attached to an animal to the sensors. The system uses these ToA estimates to estimate the location of the transmitter. Radio transmitters are used for ToA tracking of terrestrial species [2,3,4], and ultrasonic audio transmitters are used for ToA tracking of aquatic species [5,6,7,8].

The principles of ToA localization have been known for decades and the literature on modeling these estimation problems and algorithms for resolving the models is vast (see, for example, [9,10]). However, several contemporary factors combine to make the development of improved models and algorithms attractive and sometimes necessary, especially for wildlife tracking:

- New high-throughput tracking systems, which can generate millions of localizations per day across numerous individuals, often generate large numbers of incorrect estimates, even when the rate of producing incorrect estimates is low;
- Improvements in compute power mean that algorithmic approaches that were considered computationally too costly in the past are now acceptable, even for real-time localization;
- Transmitter localization wildlife tracking systems are regional by nature (their coverage is limited by the limited range of terrestrial radio receivers and underwater hydrophones), and localizations in marginal coverage areas near the edges of the system are noisier than those in central areas.

These factors led us to develop a suite of novel algorithms for reliable and statistically robust ToA location estimation, designed specifically to improve the quality of localizations produced by a popular high-throughput wildlife radio tracking system called ATLAS [4]. The algorithms were developed in response to incorrect localizations that were produced by several existing ATLAS systems. We evaluate our new algorithms using real-world data from an ATLAS system.

Our new algorithms eliminate or reduce localization failures caused by four underlying factors:

1. Time-of-arrival measurements that are inconsistent with the distance between the transmitter and the receiver, given the estimated variance of the measurement (when the estimated variance is high, the measurement is assigned a low weight and has little influence on the solution). The inconsistency can be caused by non-line-of-sight (NLOS) propagation, radio interference, glitches in the receiver’s clock, underestimation of the variance of a measurement (which is derived from an estimated signal-to-noise ratio), or software bugs.
2. ToA measurements that are consistent with two possible transmitter locations. We refer to this case as location *ambiguity*. Ambiguity usually arises when the number of constraints that define the location is equal to the number of unknown parameters, because the constraints are non-linear. The ambiguity usually disappears when the number of constraints is higher, but it can arise with any number of constraints due to symmetries.
3. ToA measurements by receivers close to the transmitter. The geometry of arrays of ToA radio receivers, which is often characterized by approximately even distribution in the plane but with very limited variation in elevation, usually does not permit an accurate estimation of the elevation of a transmitter, so its plane location is estimated given an assumed elevation. This normally leads to accurate plane estimates, except when one of the receivers is close to the transmitter (the difficulty of localizing a transmitter near one of the receivers has been known for a long time [Section III] of [3], but the reason was not understood until now).
4. Numerical failures resulting from the use of fixed-precision arithmetic (64-bit floating point numbers). Numerical failures are not common in ToA estimation algorithms, but they did prove common in the algorithm that ATLAS used to approximate the covariance of location estimates.

Section 2 describes a suite of algorithms that we developed to mitigate all of these failure cases. We used a guiding principle when developing the new algorithm: as much as possible, each localization should depend on ToA measurements of one packet transmitted by a mobile transmitter. Location samples along an animal’s track are strongly autocorrelated, so information on past and future locations strongly biases location estimates, and this is used extensively in downstream processing [11]: tracks are cleaned (localizations inconsistent with the bulk of a track are removed) and smoothed, to reflect the constraint of autocorrelated position. Our guiding principle aims to enforce modularity. At the processing phase we focus on in this paper, we aim to produce the best localization possible for each transmitted packet separately. Autocorrelation is exploited at a later processing phase. We only deviate from this principle to disambiguate ambiguous localization problems; here, the only way to select the correct choice is to use information from unambiguous future and/or past localizations.

The main contributions of this articles are:

- New methods to detect and discard outlier ToA measurements, which can be caused by non-line-of-sight propagation, radio interference, clock glitches, or an overestimation of the signal-to-noise ratio.
- A method to detect cases in which two locations are equally consistent with the measurements and to select the correct one in most cases.
- A method to infer approximate altitude information from a digital elevation map, which improves location estimates close to one of the sensors.
- A new method to approximate the covariance matrix of location estimates. The new method is simpler and more reliable than the baseline algorithm.
- Integration of all these new methods into high-performance production-quality implementations.
- Demonstration of the efficacy of the new algorithms using extensive testing on real-world data involving both fixed and mobile transmitters, some attached to wild animals.
- Demonstration of the performance of the new algorithms, allowing them to be used in near-real-time high-throughput wildlife localization systems.

### 1.1. ATLAS

ATLAS has proven to be a maintainable and productive regional high-throughput wildlife tracking system. It can track many species of wild animals at high temporal and spatial resolution using ToA radio transmitter localization [3,4,12,13,14]. Six separate ATLAS systems, each consisting of 5 to 25 receivers, in four countries have tracked over 7000 transmitters, called *tags*, over the past 8 years, and a few more systems are under construction. Some of these systems consist of more than 25 receivers and cover more than $1000\;{\mathrm{km}}^{2}$ [13]. Tracking of fruit bats [15], insect-eating bats [16], pheasants [17], barn owls [18], shorebirds [13], and avian predators [19] by these systems has led to several research articles in the scientific (ecology) literature.

### 1.2. System Model and Time-of-Arrival Constraints

ATLAS systems localize mobile radio transmitters, or tags, by estimating the time of arrival of transmitted packets to receivers, also called *base stations*, at known locations. We denote the unknown location $\ell_{i}=\left[\begin{array}{ccc} x_{i} & y_{i} & z_{i} \end{array}\right]$ of a tag that transmits a packet at an unknown time as $\tau_{i}$. Transmission times are periodic, but the periodicity is not accurate enough to be useful for radio time-of-arrival localization. We denote the known location of receiver *r* by $\rho_{r}$. The fundamental equation that relates the time of transmission $\tau_{i}$ to the exact absolute time ${\overset{˚}{t}}_{ir}$ of which the packet arrives at the antenna of receiver *i* is

(1) $${\overset{˚}{t}}_{ir}=\tau_{i}+\frac{1}{c}{∥\rho_{r}-\ell_{i}∥}_{2}\;,$$

where *c* is the known speed of propagation (speed of light). The receiver estimates the time of arrival of the packet at the digital part of the receiver. The estimate, denoted $t_{ir}$, is related to the time of transmission and to the locations by the following equation,

(2) $$t_{ir}=\tau_{i}+\frac{1}{c}{∥\rho_{r}-\ell_{i}∥}_{2}+o_{r}+\epsilon_{ir}\;,$$

where $o_{r}$ is the sum of the delay of the signal from the antenna to the digital part of the receiver and the offset of the clock of the receiver, and $\epsilon_{ir}$ is the time-of-arrival estimation error. From here on, we refer to $o_{r}$ simply as the offset of receiver *r*, even though it is the sum of a delay and an offset. The error $\epsilon_{ir}$ is random and is not known, but the receiver estimates its variance from an estimate of the signal-to-noise ratio (SNR) of the packet [4]. We denote the estimated variance by $\sigma_{ir}^{2}$. The arrival time estimation errors of the same packet in different receivers are probably sometimes correlated, but ATLAS treats them as uncorrelated.

We treat $o_{r}$ as a function of the receiver *r* and not as a function of time because ATLAS receivers use GPS-disciplined clocks with highly accurate rates.

Because the equations include the sum $\tau_{i}+o_{r}$ we cannot resolve both. If $\tau_{i}$ and $o_{r}$ satisfy the equations, so do $\tau_{i}+\alpha$ and $o_{r}-\alpha$, for any real α. Therefore, ATLAS arbitrarily fixes one of these quantities to zero. This effectively selects a particular reference clock; the choice has no effect on the estimation of $\ell_{i}$.

We refer to known quantities in Equation (2) as a *detection* (a short form of *detection report*) and we refer to a set of detections of the same packet as a *detset* (short form of *detections set*).

The location of the tag cannot be estimated from one detset, because a detset of size *m* represents *m* equations of the form (2) with m+3+1−1 unknowns: the *m* offsets, the three coordinates of $\ell_{i}$, and the time of transmission $\tau_{i}$, but minus the quantity that is fixed to zero. To be able to estimate the unknown location, ATLAS associates with each detset of a mobile transmitter one or more detsets of transmitters at known locations $\ell_{b}$. We refer to these transmitters as *beacons* (in underwater ultrasonic ToA localization, beacons are called *sync tags*). The beacon equations are

(3) $$t_{br}=\tau_{b}+\frac{1}{c}{∥\rho_{r}-\ell_{b}∥}_{2}+o_{r}+\epsilon_{br}\;.$$

If the same *m* receivers receive both the tag and a beacon packet, they produce together 2m equations in m+3+1 unknowns. We can solve the equations when m≥4. With m=4, there is usually an exact solution satisfying $\epsilon_{ir}=0$ and $\epsilon_{br}=0$. With m>4, ATLAS solves the equations in the weighted least-squares sense, minimizing $\sum \epsilon_{ir}^{2}/\sigma_{ir}^{2}+\sum \epsilon_{br}^{2}/\sigma_{br}^{2}$. ATLAS can also associate detsets of multiple beacon packets $b,b^{\prime },b^{\prime \prime },\ldots$ with a single mobile transmitter packet and use all of them; this adds information about the offsets and can improve (usually only slightly) the accuracy of the resulting location estimate.

The elevations of ATLAS receivers do not vary much, so ATLAS systems usually cannot accurately estimate the altitude of tags (their *z* coordinate). Therefore, virtually all the localizations that ATLAS systems have estimated so far have been estimated in an *assumed-altitude* mode (2D mode for short), in which a known value was assumed for the *z* coordinates. More specifically, the value that was used was the mean elevation of the terrain in the region in which the system is deployed. In the assumed-altitude mode, tag detsets have m+2 unknowns, problems with m=3 receivers can be solved exactly, and problems with more than three receivers are solved in the least-squares sense. Unless otherwise noted, we assume from here on that locations are estimated in 2D mode.

### 1.3. Data Flow and Information Processing

ATLAS receivers place radio frequency (RF) samples in a cyclic buffer. Samples are typically processed a few seconds or less after being received. In most cases, detection reports are sent via the internet to the ATLAS server with a delay of a few seconds relative to the actual arrival time. When there is no network connection between a receiver and the server, the receiver buffers the data and uploads them to the server when connection is reestablished.

The server collects detections in a data structure called a *detset grouper*. Its job is to group together detections of each transmitted packet. The default grouper relies on an approximate time stamp associated with each detection; these are accurate to within about 1 ms. To ensure that detections are grouped correctly, detections linger in the grouper for up to 30 s to compensate for varying network delays.

Grouped detsets are handed over to a *localization problem generator*. Its job is to group together a detset of a mobile tag with several detsets of beacons from around the time of the tag’s transmission. In order to be able to use beacon transmissions that follow the tag’s, the generator delays tag detsets for another few seconds, to ensure that all relevant beacon detsets are available.

Generated localization problems are solved by a software object called the *localization server*. The resulting localizations are typically stored in a database and are sometimes also fed to real-time clients (e.g., a real-time web application).

The total delay between a tag’s transmission and the estimation of its location is about 45 s.

ATLAS estimates locations not only in real time, but in two additional settings, in which detections are read in time order from a database.

First, a few minutes after the beginning of a round hour, ATLAS recomputes all the localization for the preceding hours. The process is repeated if a receiver uploads detections that are hours or days old, following a disconnected period. This setting is called the *hourly consistency enforcement*. It can use more expensive and complex algorithms than the real-time setting.

Finally, ATLAS users also compute location estimates in a batch query. This is typically performed in order to compute the localizations using algorithmic parameters that are different from the parameters that were used to compute the localizations stored in the ATLAS database.

**Figure 1:**
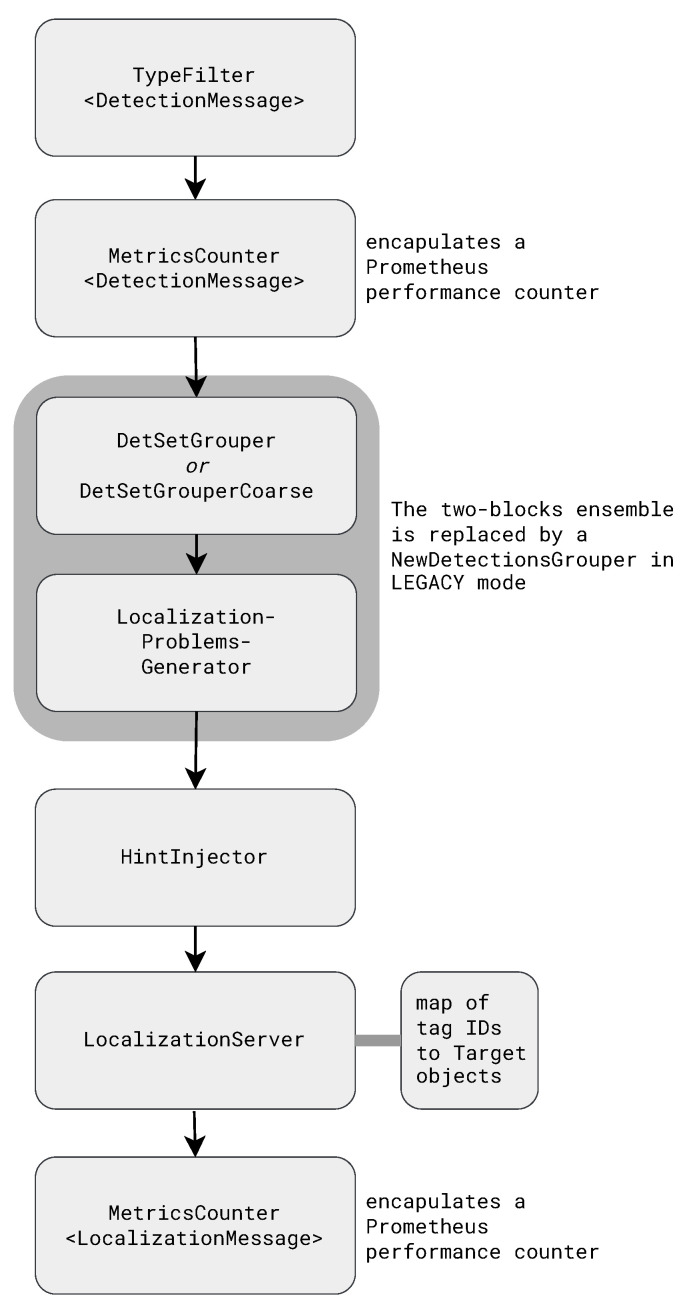
Data flow in the localization subsystem. The topmost processing block accepts a stream of detections and the block at the bottom generates a stream of localizations.

Figure 1 shows the data flow in the localization subsystem of an ATLAS server. The key processing steps are the grouping of detections into detsets, the generation of localization problems, which consist of a tag detsets and a few beacon detsets from about the same time, hint injection, and estimating locations.

### 1.4. From Location Estimation Problems to Objective Functions

Most modern location estimation systems estimation locations by minimizing a non-linear function of the observations [9,20,21].

Localization problems produced by the generator consist of a tag detset and several beacon detsets from nearby times. The localization server transforms each problem into one or more objective functions objects. The objective functions are scalar functions of the unknown parameters, defined such that the best estimate lies at a local minimum of the function. More specifically, ATLAS, like most other location estimation systems, defines least-squares objective functions [9,21,22]. The most straightforward functions have the form

$$\begin{array}{ccc} g\left(\ell_{i},\tau_{i},\tau_{b},o_{r}\right)\;= & \sum_{r} & \epsilon_{ir}^{2}+\epsilon_{br}^{2}\\ & =\sum_{r} & {\left(t_{ir}-\tau_{i}-\frac{1}{c}{∥\rho_{r}-\ell_{i}∥}_{2}-o_{r}\right)}^{2}+\\ & & {\left(t_{br}-\tau_{b}-\frac{1}{c}{∥\rho_{r}-\ell_{b}∥}_{2}-o_{r}\right)}^{2}\;. \end{array}$$

The representation of the objective function is an object that can evaluate its value and its derivatives, and in particular the matrix of its first derivatives, called the Jacobian.

The class of algorithms that are considered best for solving non-linear least squares problems are iterative algorithms from the Gauss–Newton family, such as the Levenberg–Marquardt algorithm [23]. By iterative, we mean that these algorithms produce a sequence of estimates, starting from some initial guess, which hopefully converges to an accurate one.

The success rate and the performance of iterative non-linear least-squares solvers depend on both the dimension of the problem (the number of unknown parameters) and on the quality of the initial guess. Initial guesses near the true solution lead to rapid convergence to an accurate estimate; far away guesses require more iterations and can sometimes lead to convergence to a wrong local minimum, or to divergence. Problems in high dimensions are harder.

ATLAS addresses the dimension challenge by exploiting of the separability of systems of constraints of the forms (2) and (3) and eliminates the unknowns that the prediction depends on linearly, line $\tau_{i}$, $\tau_{b}$, and $o_{r}$, using a one-shot matrix operation [4]. Therefore, the representation of the objective function in ATLAS takes only a location hypothesis $\ell_{i}$ and produces

$$f\left(\ell_{i}\right)=\min_{\tau_{i},\tau_{b},o_{r}}f\left(\ell_{i},\tau_{i},\tau_{b},o_{r}\right)$$

and the Jacobian of *f*.

ATLAS supports three types of objective functions of this type: single-beacon, multiple-beacons, and differenced or simplified, explained below. The localization server can construct these functions in two or three dimensions (the dimension specifies the number of unknown parameters in $\ell_{i}$; distances are always evaluated in 3D).

The same objective functions are used by the old, baseline algorithm in ATLAS and by the new robust algorithms. However, in the new algorithms, data from some receivers is sometimes dropped from an objective function.

### 1.5. ATLAS’s Baseline Localization Algorithm: Generating and Ranking Initial Guesses

ATLAS’s baseline algorithm [4], the one that we aim to improve, is essentially a sequence of attempts to find a good initial guess, followed by an invocation of the Levenberg–Marquardt algorithm to compute the final maximum-likelihood estimate. The sequence includes a fixed point in the center of the coverage area, the previous estimated location of the tag, invocation of a derivative-free optimization algorithm [24], Nedler–Mead [25], a simple grid search, and invocations of Levenberg–Marquardt on simplified objective functions (2D rather than 3D or using a single beacon rather than multiple ones). In this sequence, the initial guess provided to iterative algorithms is the best estimated found up to that point in the sequence.

Clearly, for this strategy to work, the algorithm requires a mechanism to assess the quality of a location estimate. The assessment is used in two ways. First, it is used to rank location estimates, so as to start the next iterative solver in the sequence (or the ultimate one) from the best estimate found so far. Second, it is used to decide whether to skip expensive steps in the sequence. For example, if the previous location of the tag is a good estimate for the current location, we can skip the entire sequence and invoke Levenberg–Marquardt on the ultimate objective function. Similarly, if Nedler–Mead found a reasonable estimate, we can skip the expensive grid search.

The score that ATLAS’s baseline algorithm uses to assess the quality of estimates is the maximum norm of the residual of a simplified 2D single-beacon objective function. The norm is expressed in meters, not nanoseconds, for clarity. The success rate of ATLAS’s baseline algorithm depends critically on the robustness of this objective function. Since no attempt was made to remove outliers from it, it has not always been robust.

### 1.6. Approximating the Covariance of Location Estimates

ATLAS’s baseline algorithm [4] approximates the covariance matrix of computed localizations using a formula that was derived using the implicit-function theorem, specialized to non-linear least squares. The formula requires the evaluation of second derivatives of the function that predicts the observed quantities. The authors of [4] developed analytic expressions for these derivatives. The expressions involved squaring and cubing the distance between tags and receivers. This has led to numerical failures, including ones in which ATLAS presented localizations with large errors as having tiny variances.

### 1.7. Related Work

We review in this subsection related work on statistically robust location estimation and on closed-form solvers for ToA location estimation. For a thorough survey of robust wireless location estimation, see the survey by Güvenç and Chong [22]. We also note that several efforts to improve ultrasonic underwater tracking systems, some specialized to aquatic wildlife tracking, have also been reported in the literature [5,26,27].

Our outlier identification method is based on RANSAC [28]. However, RANSAC cannot be applied naively to our problem, primarily because the number of observations in each problem is typically too small. Li et al. [29] presented a robust detect-and-discard method to localize a target using distance measurements. Our consensus-based outlier detector is closely related to their method. They selected random subsets of slightly over-determined constraints and solved them using a combination of a closed-form approximation and an iterative solver; this gives them a set of hypotheses. They then computed a full residual vector for each hypothesis, selected the one in which the median of the squared residual elements is smallest [30], and used it to identify outliers. Once outliers were removed, they solved the remaining constraints in the least-squares sense; we perform the same. Our method improves upon their in three ways. First, their closed-form solver only finds one solution for each subset of constraints, but there can be two; ours finds both. Second, their use of the minimum median residual element is useless when there are not very many constraints (three constraints can always be satisfied exactly, so when there are at most six constraints, the median is always zero and carries no information). Third, their choice of subsets in completely random; we bias the selection towards constraints with high-confidence measurements.

Kung et al. [31] follow a different approach to robust location estimation. Vector norms that are less sensitive to large residual elements than the 2-norm, like the 1-norm, tolerate moderate outliers better. Kung et al. [31] propose a parameterized family of specialized penalty functions, called SISR, that are designed to give little weight to outliers and high weight to inliers; they use them, sometimes with re-parameterization, to estimate locations from distance measurements. In general, such methods do not cope well with gross outliers, and they are computationally more expensive than methods based on least-squares building blocks like ours and like that of Li et al. [29]. Park and Chang [32] propose another method using the same general approach.

Liu et al. [33,34] propose yet another outlier identification method for localizing multiple targets from distance measurements. Their main idea is to identify the worst outlier by solving least-squares problems involving all but one constraint and repeat. This avoid the combinatorial explosion of RANSAC in high dimensions but suffers from the problem that RANSAC aims to avoid, namely the fact that least-squares solutions that include outliers are often completely useless for identifying outliers.

So-called harsh environments can lead to more than 50% outliers. Yin et al. [35] propose a wireless ToA localization method that can cope with such environments. Since ATLAS is used in relatively open areas, the fraction of outliers in ATLAS systems is much smaller.

Recently, machine learning and neural networks are beginning to be explored for robust localization [2,26]. In some cases, machine learning is used to classify outliers and in others to also estimate locations from ToA or differences of ToA measurements. These approaches require training for a particular geography, which is not necessary in our approach. Some of these approaches (e.g., [2]) also ignore physical and mathematical constraints that our approach exploits, such as the statistical relationship between the signal-to-noise ratios and ToA measurement errors.

Closed-form solvers for these types of constraints have been known for many years [36], but to the best of our knowledge, were always seen as a fast non-iterative algorithm for finding an approximate location estimate (see, e.g., [37]). For this purpose, they are not particularly attractive [38]. In contrast, We use the solver described below not to generate location estimates, but to quickly generate hypotheses, as well as to enumerate the two solutions of an exactly determined problem so that we can disambiguate them. Our method is not new, but the derivation and presentation are new and completely algebraic. They should be easier for most readers to follow and implement than equivalent presentations in the literature [36,38].

## 2. Materials and Methods

We now present the new algorithms that are the main focus of this paper. The new algorithms use the same objective functions as the baseline algorithm (but sometimes with only a subset of the constraints), but they make more conservative assumptions and a completely different approach.

More specifically, the new algorithms generate the initial guess for the iterative minimization of the ultimate objective function not using a sequence of algorithms, but by enumerating hypotheses that arise from closed-form solutions of minimal subsets of constraints (see Section 2.9 below), and by selecting the best hypothesis either using a consensus-based technique, inspired by the RANSAC method [28], or by using a clustering technique.

The new algorithms also ignore constraints that appear to be highly inconsistent with a reasonable hypothesis; this is also inspired by RANSAC.

The new algorithms also recognize that some location estimation problems are highly likely to have two solutions with low residuals (good consistency with the constraints) and it treats such problems cautiously, aiming to enumerate the solutions and to choose the right one based on high-confidence localizations of the tag shortly before or after the current problem.

The algorithms have been implemented in Java. The Apache Commons Math library was used for matrix computations and for the clustering algorithm. The code is single-threaded.

### 2.1. Classifying Outliers

One of the most important advances in the new release is a mechanism to identify outlier detections, so they can be removed from the least-squares problem whose solution constitutes the location estimate. The classification of outliers is always performed with respect to a *hypothesis*, either a location-only hypothesis ${\bar{\ell }}_{i}=\left[\begin{array}{ccc} {\bar{x}}_{i} & {\bar{y}}_{i} & {\bar{z}}_{i} \end{array}\right]$, or a location-and-time hypothesis ${\bar{\ell }}_{i}$, ${\bar{d}}_{ir}$ where ${\bar{d}}_{ir}$ is an estimate of the transmission time difference $\tau_{i}-\tau_{b}$.

We classify outliers using difference constraints obtained from subtracting Equation (3) from Equation (2),

$$\begin{array}{ccc} t_{ir}-t_{br} & = & \tau_{i}+\frac{1}{c}{∥\rho_{r}-\ell_{i}∥}_{2}+o_{r}+\epsilon_{ir}\\ & - & \tau_{b}+\frac{1}{c}{∥\rho_{r}-\ell_{b}∥}_{2}+o_{r}+\epsilon_{br}\\ & = & \left(\tau_{i}-\tau_{b}\right)+\frac{1}{c}{∥\rho_{r}-\ell_{i}∥}_{2}-\frac{1}{c}{∥\rho_{r}-\ell_{b}∥}_{2}+\left(\epsilon_{ir}-\epsilon_{br}\right)\;, \end{array}$$

or

(4) $$t_{ir}-t_{br}+\frac{{∥\rho_{r}-\ell_{b}∥}_{2}}{c}=\left(\tau_{i}-\tau_{b}\right)+\frac{{∥\rho_{r}-\ell_{i}∥}_{2}}{c}+\left(\epsilon_{ir}-\epsilon_{br}\right)\;.$$

The differencing eliminates the unknown offset $o_{r}$. It is possible to show that if the covariance matrix of the time-of-arrival estimation errors ϵ is diagonal with diagonal elements $\sigma_{ir}^{2}$, $\sigma_{br}^{2}$, then the weighted least-squares solution of the original constraints (2) and (3) is the same as the weighted least-squares solution of the difference constraints (4), if they are weighted by $\sqrt{\sigma_{ir}^{2}+\sigma_{br}^{2}}$ [Section 10.4] of [20]. To simplify the notation, we denote the difference quantities $t_{ibr}=t_{ir}-t_{ibr}$, $\tau_{ib}=\tau_{i}-\tau_{b}$, and $\sigma_{ibr}=\sqrt{\sigma_{ir}^{2}+\sigma_{br}^{2}}$.

We classify outliers using a hypothesis and a weighted residual threshold. Given a location and time hypothesis ${\bar{\ell }}_{i},{\bar{d}}_{ir}$ and a threshold δ, we classify as outliers all the pairs $t_{ir},t_{br}$ for which

(5) $$\left|t_{ir}-t_{br}+\frac{{∥\rho_{r}-\ell_{b}∥}_{2}}{c}-{\bar{d}}_{ir}-\frac{{∥\rho_{r}-{\bar{\ell }}_{i}∥}_{2}}{c}\right|>\frac{\delta }{\sigma_{ibr}}\;.$$

In other words, we consider differenced measurements that deviate from the hypothesis-based prediction by more than δ standard deviations to be outliers. If we only have a hypothetical location ${\bar{\ell }}_{i}$, we first produce a robust value

$${\bar{d}}_{ir}={\mathrm{median}}_{r}\left(t_{ir}-t_{br}+\frac{{∥\rho_{r}-\ell_{b}∥}_{2}}{c}-\frac{{∥\rho_{r}-{\bar{\ell }}_{i}∥}_{2}}{c}\right)$$

for $\tau_{i}-\tau_{b}$ and then apply the same rule.

### 2.2. Hypotheses, Consensus Sets, Disambiguation, and Selection

Two important innovations in the new algorithms are (1) the algebraic generation of location hypotheses and (2) mechanisms to select a good hypothesis, along with a single-beacon problem consisting only of constraints that are in agreement (consensus) with the hypothesis. We then solve this problem starting from the hypothesis; the solution is a good initial guess for the ultimate solver.

We produce hypotheses in two ways. Given detest *i* for a tag and all the detsets of beacons with $|\tau_{ib}|$ of up to a few seconds, we first produce a set of rough *closed-form hypotheses*. This happens in two phases. First, we rank the beacon detsets. The ranking is lexicographic, starting with the cardinality of the intersection with detset *i* (number of receivers that received both the tag and the beacon), then approximate time difference $\tau_{i}-\tau_{b}$ less than 2 s (we use $t_{ir}-t_{ibr}$ for some *r* as the approximation), and finally the SNR of the beacon detections in the intersection.

Next, we generate hypotheses from a few of the highest ranked beacon detsets (by default from the best five). For each admitted beacon detset, we form the differenced constraints (4) and generate a few triplets of constraints, ordered by largest $\sigma_{ibr}$ in the triplet. That is, we favor triplets of constraints with small standard deviations. Each triplet gives us three constraints in three unknowns. We then use a specialized algebraic closed-form solver to analytically solve each triplet. Geometrically, the solutions are intersections of two half-hyperbolas in the plane. Normally each triplet produces two solutions with zero residuals, but there could be only one solution or no solutions at all. These are the rough closed-form hypotheses; they consist of both a location ${\bar{\ell }}_{i}$ and a time difference ${\bar{d}}_{ir}$.

From here on, we select the best hypothesis in one of two ways.

**Figure 2:**
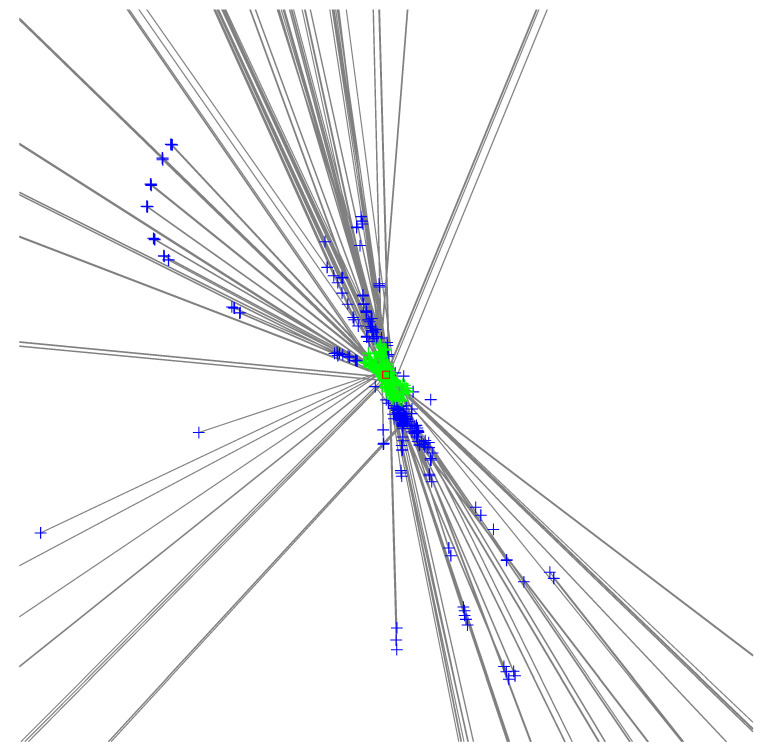
Data from a run of the clustering algorithm. Each plus sign represents a hypothesis, one solution of a closed-form problem. Two solutions of the same problem are connected by a gray line. The hypotheses in the largest DBSCAN cluster are depicted in green, the rest in blue. The hypothesis closest the medians of the cluster is marked by a red square. The figure was cropped to 5 by 5 km; the hypotheses spanned an area of 3723 by 17,204 km. The largest cluster consisted of 299 hypotheses out of 726 and it spanned 0.3 by 0.4 km. The localization problem consisted of detections from 16 base stations and is one of the problems used in Table 1.

One variant, which we refer to as the *clustering* variant, is based on the observation that in the absence of outliers, each triplet produces one solution near the target’s location. We run a clustering algorithm, DBSCAN [39], on the set of all hypotheses locations (that is, only on the ${\bar{\ell }}_{i}=\left[\begin{array}{cc} x_{i} & y_{i} \end{array}\right]$ coordinates of the solutions, discarding the ${\bar{d}}_{ir}$s), find the largest cluster, compute a center for the cluster, and then return the solution closest to this center point. We define the center as the median of the *x* coordinates and the median of the *y* coordinates in the cluster. The process is illustrated in Figure 2 using real-world data. Because we return an actual solution, we also have its ${\bar{d}}_{ir}$ value, and we know which single-beacon transmission it came from. The parameters of this variance are set so as to produce many hypotheses, up to a few hundreds; this makes the clustering robust.

The other variant, which we refer to as the *consensus* variant, tests all the constraints of a single-beacon problem for consistency, in the sense of Equation (5), with a hypothetical solution (one of the closed-form solutions). We rank the hypotheses lexicographically by the number of consistent constraints (the size of the so-called *consensus set*), then by the distance to a prior estimate (as long as the largest weighted residual in the consensus sets differ by at most 1), and then by the largest weighted residual.

In both variants, we now have a hypothesis and the single-beacon transmission from which the hypothesis was generated. If the consensus set of the best closed-form hypothesis contains more than three constraints, we improve this hypothesis by solving all the constraints in the consensus set in the weighted least-squares sense using an iterative solver, starting from the selected hypothesis. We refer to the solution as a *single beacon-transmission hypothesis* ${\bar{\bar{\ell }}}_{i}$. It differs from the closed-form hypotheses in that it is based on a least-squares solutions of all the constraints of the consensus set, not only three.

### 2.3. Building the Ultimate Set of Constraints

The user chooses whether to estimate $\ell_{i}$ using multiple beacon transmissions or only one. Either way, we estimate $\ell_{i}$ from a set of constraints of the form (2) or (3), not from the difference constraints (4). This set is the union of the constraints that make up the inlier difference constraints in one or more single beacon-transmission problems.

If the user chooses to use one beacon transmission, we use inliers from the best hypothesis.

If the user chooses to use multiple beacon transmissions, we classify the difference constraints in all the single-beacon transmission problems using ${\bar{\bar{\ell }}}_{i}$ and use the constituent constraints of all the inliers.

### 2.4. Altitudes, Four Ways

The user also specifies how to treat the altitude of the tag. There are four options. First, the system can assume a fixed height, say 0 for coastal systems tracking shore birds, or average ground level plus 30 m for systems tracking birds or bats over flat terrain. In this case, the ultimate set of constraints is solved once with the altitude treated as a known quantity. This approach is suitable for very flat regions.

Second, the system can assume that the tag is at a fixed height above the terrain, say 0 m for a pheasant or 30 m for a bat. In this case, the ultimate set of constraints is solved twice. The first solution sets the altitude to a fixed value. A digital elevation model (DEM) is then used to determine the elevation at the estimated *x*-*y* coordinates. We now solve the constraints again, constraining the altitude to this elevation (or to a fixed offset from it). This is a good solution for more hilly terrain and for animals that spend most of the time on the ground or at low altitude above ground. DEM files with resolution of about 30 m are freely available for most of the Earth [40].

Third, the system can use the altitude sensed by an air pressure altimeter [41]. This is the most accurate option but requires either data upload from the tag to a base station or recapture of the animal to retrieve the tag to collect stored data.

Finally, the system can simply estimate all three coordinates of $\ell_{i}$ from time-of-arrival measurements if the tag was received by at least four receivers. Because the elevation of receivers typically does not vary much, the altitude estimate produced by this method often has a high variance, and errors in the altitude estimate can degrade the *x*-*y* location estimate, so this is a technical capability that is usually not useful for wildlife tracking.

### 2.5. Representation and Maintenance of a Prior Distribution

Our new algorithms maintain a state-space representation of tag tracks. This representation is treated as a prior distribution of the location of a tag at the time of transmission. The representation consists of the location and optionally the velocity of the tag, an estimate of the covariance matrix of these quantities, as well as a binary flag that aims to differentiate between ambiguous states and unambiguous states.

This prior distribution is used in the ranking of the hypotheses in the consensus variant. The code can use either the density of the prior distribution, which takes into account the shape of the covariance matrix, or just the distance to the prior location estimate; we currently use only the latter, which proved more robust in preliminary evaluations. The prior distribution can also be used as the initial guess in the legacy algorithm.

The numerical part of the state is normally advanced using a Kalman filter. Each localization of a tag constitutes a set of observation constraints on the location of the tag, weighted by the estimated covariance of the localization. The state of the filter is advanced using a system of evolution constraints. The user can choose between evolution constraints that assume constant position and evolution constraints that assume constant velocity. The covariance matrix of the evolution constraints is set so as to correctly reflect the dynamics of an animal. For example, in the constant-position constraints, it makes sense to set the standard deviations of the constraints to about 10 m times the number of seconds between localizations, reflecting the prior knowledge that the animal can move at about 10 m/s but not much faster. To use the state of the Kalman filter as a prior distribution for the next location estimate, the state of the filter is advanced and the resulting state is used as the prior, not the state at the last observation.

New localizations advance the state of the Kalman filter only if they are reasonably consistent with the current state. Inconsistency is defined by distance larger than $20\Delta_{t}$ where $\Delta_{t}$ is the time difference, or distance of more than 250 m if the localization is based on only three base stations, or a distance of more than 1 km if the localization is based on four base stations. This outlier rejection mechanism proved highly important for the algorithm.

The binary flag starts in the ambiguous state; it also reverts to the ambiguous state once the last localization of the tag is older than 20 min. The flag transitions to the unambiguous state if the tag is localized from detections by six or more receivers, or if it was recently localized at least five times from detections of four or more base stations (four or more each time). In the latter case, the state is set to localization among the five whose coordinates are the closest to the medians of the five locations in the *x* and the *y* directions, for robustness.

### 2.6. Revisiting Ambiguous Location Estimates

When a tag is detected for the first time or detected again after a long absence, and it is detected by only three receivers, we often cannot disambiguate between two closed-form hypotheses. The detections from the three receivers are often exactly consistent with two separate locations. In some cases, one of the two locations will be so far from the receivers that we know that the tag could not have transmitted from this location, but often both locations are plausible.

When the algorithm is used for real-time localization, we emit one of the locations so that the user knows that the tag can be localized. The user can inspect the localization and see that it is ambiguous. In principle, we also can emit both locations, but we do not think that this would be useful to users.

However, ATLAS always recomputes locations at the end of each round hour, and users can also ask the system to recompute data on demand. In these batch offline computations, the system tries to refrain from emitting ambiguous localizations.

This mechanism has several algorithmic components. The first is the classification of location estimates into ambiguous and unambiguous ones. We classify an estimate as ambiguous if it was produced from time-of-arrival constraints of only three receivers when the prior distribution was also labeled as ambiguous.

In the offline batch setting, ambiguous estimates are not emitted; instead, all the raw data are stored and is re-processed later. In most cases, the system is able to compute an unambiguous location estimate for the tag at some later time, when the tag is detected by four or more receivers in consensus. When this happens, the system propagates unambiguous location estimates backward in time all the way to the beginning of the queue of ambiguous problems.

The number of ambiguous localization problems that the system stores in memory for each tag is capped. When the limit is reached, the system recomputes an estimate of the oldest stored problem and emits it, even though it is ambiguous.

### 2.7. Providing Hints

When the system starts a batch localization process using detections stored in the database, it initializes the prior distribution from localizations stored in the database. More specifically, it retrieves from the database the 10 most recent localizations based on four or more base stations of each tag and uses them to initialize the prior distribution of the tag. Only localizations from the 12 h prior to the start time of the batch are used.

### 2.8. Estimating the Covariance of a Location Estimate

We also improved the estimation of the covariance of location estimates. Rather than trying to fix the numerical issues in the original algorithm, we developed a new covariance approximation that is simpler and only requires first derivatives, not second derivatives.

The final location estimate $\hat{\ell }$ is always a least-squares minimizer,

$$\hat{\ell }=f\left(b\right)=\arg\min_{\ell }{∥B\left(M\left(\ell \right)-b\right)∥}_{2}^{2}\;,$$

where *M* is a function that maps hypothetical solutions *ℓ* to the measured quantities (arrival times or arrival-time differences), *b* consists of the actual measurements, and *B* is a composition of a projection that eliminates nuisance parameters (transmission times and clock offsets) and a weighting matrix [4].

For a small perturbation δ of the observations we have

$$f\left(b+\delta \right)=f\left(b\right)+J_{f}\left(b\right)\delta +O\left({∥\delta ∥}^{2}\right)\approx \hat{\ell }+J_{f}\left(b\right)\delta \;.$$

We denote by $f\left(b+\delta \right)=\hat{\ell }+\hat{\Delta }=\arg\min_{\ell }∥B\left(M\left(\ell \right)-\left(b+\delta \right)\right)∥$. Our strategy is to approximate $J_{f}\left(b\right)$ by expressing $\hat{\Delta }$ as a linear transformation of δ; that linear transformation approximates $J_{f}\left(b\right)$. We define $\Delta =\ell -\hat{\ell }$ or $\ell =\hat{\ell }+\Delta$, so a first-order approximation of M(ℓ), and a change in variables leads to

$$\begin{array}{ccc} \hat{\ell }+\hat{\Delta } & = & \arg\min_{\ell }{∥B\left(M\left(\ell \right)-\left(b+\delta \right)\right)∥}_{2}^{2}\\ & \approx & \arg\min_{\ell }{∥B\left(M\left(\hat{\ell }\right)+J_{M}\left(\hat{\ell }\right)\left(\ell -\hat{\ell }\right)-\left(b+\delta \right)\right)∥}_{2}^{2}\\ & = & \hat{\ell }+\arg\min_{\Delta }{∥B\left(M\left(\hat{\ell }\right)+J_{M}\left(\hat{\ell }\right)\Delta -\left(b+\delta \right)\right)∥}_{2}^{2}\;. \end{array}$$

The second term is a linear least-squares problem whose solution is

$$\begin{array}{ccc} \hat{\Delta } & = & \arg\min_{\Delta }{∥B\left(M\left(\hat{\ell }\right)+J_{M}\left(\hat{\ell }\right)\Delta -\left(b+\delta \right)\right)∥}_{2}^{2}\\ & = & {\left(BJ_{M}\left(\hat{\ell }\right)\right)}^{+}\left(BM\left(\hat{\ell }\right)-B\left(b+\delta \right)\right)\\ & = & -{\left(BJ_{M}\left(\hat{\ell }\right)\right)}^{+}B\delta +{\left(BJ_{M}\left(\hat{\ell }\right)\right)}^{+}B\left(M\left(\hat{\ell }\right)-b\right)\\ & = & -{\left(BJ_{M}\left(\hat{\ell }\right)\right)}^{+}B\delta \;. \end{array}$$

We dropped the rightmost term in the line before last because it is zero. To show that we note that the gradient of *f* is zero at the minimizer $\hat{\ell }$,

$$\begin{array}{ccc} 0 & = & \nabla_{f}\left(\hat{\ell }\right)\\ & = & 2{\left(BM\left(\hat{\ell }\right)-Bb\right)}^{T}\frac{\partial BM(\hat{\ell })}{\partial \ell }\\ & = & 2{\left(BM\left(\hat{\ell }\right)-Bb\right)}^{T}BJ_{M}\left(\hat{\ell }\right)\;. \end{array}$$

Therefore,

$$\begin{array}{ccc} {\left(BJ_{M}\left(\hat{\ell }\right)\right)}^{+}B\left(M\left(\hat{\ell }\right)-b\right) & = & {\left(J_{M}^{T}\left(\hat{\ell }\right)B^{T}BJ_{M}\left(\hat{\ell }\right)\right)}^{-1}\frac{1}{2}\nabla_{f}^{T}\left(\hat{\ell }\right)\\ & = & 0\;. \end{array}$$

We have shown that

$$\hat{\Delta }=-{\left(BJ_{M}\left(\hat{\ell }\right)\right)}^{+}B\delta \;,$$

so

$$J_{f}\left(b\right)\approx -{\left(BJ_{M}\left(\hat{\ell }\right)\right)}^{+}B\;.$$

When we take cov(ϵ) to be diagonal, which is the case in practice [4], we can simplify the expression for the covariance of $\hat{\ell }$ using the structure of $B=(I-UU^{T})W$, where *U* is orthonormal and $WW^{T}={\mathrm{cov}}^{-1}\left(\epsilon \right)$. We have

(6) $$\begin{array}{ccc} \mathrm{cov}\left(\hat{\ell }\right) & \approx & J_{f}\left(M\left(\hat{\ell }\right)\right)\mathrm{cov}\left(\epsilon \right){\left(J_{f}\left(M\left(\hat{\ell }\right)\right)\right)}^{T}\\ & = & {\left(BJ_{M}\left(\hat{\ell }\right)\right)}^{+}B{\left(W^{2}\right)}^{-1}B^{T}{\left({\left(BJ_{M}\left(\hat{\ell }\right)\right)}^{+}\right)}^{T}\\ & = & {\left(BJ_{M}\left(\hat{\ell }\right)\right)}^{+}\left(I-UU^{T}\right){\left({\left(BJ_{M}\left(\hat{\ell }\right)\right)}^{+}\right)}^{T}\;. \end{array}$$

The expression on the last line is the one we use as an estimate of $\mathrm{cov}(\hat{\ell })$.

### 2.9. An Algebraic Closed-Form Solver

We now show how to find closed-form solutions to exactly determined differenced time-of-arrival localization problems (2D problems with m=3 or 3D problems with m=4).

To keep the notation in this section simple, we rename the quantities in (4) as follows:

$$\begin{array}{ccc} c\left(t_{ir}-t_{br}\right)+{∥\rho_{r}-\ell_{b}∥}_{2} & \mapsto & t_{i}\\ c\left(\tau_{i}-\tau_{b}\right) & \mapsto & t\\ \rho_{r} & \mapsto & \left[\begin{array}{ccc} x_{i} & y_{i} & z_{i} \end{array}\right]\\ \ell_{i} & \mapsto & \left[\begin{array}{ccc} x & y & z \end{array}\right]\;. \end{array}$$

Consider a system of equations of the form

(7) $$t_{i}-t=\sqrt{{\left(x_{i}-x\right)}^{2}+{\left(y_{i}-y\right)}^{2}+{\left(z_{i}-z\right)}^{2}}$$

where the $t_{i}$s, $x_{i}$s, $y_{i}$s, and $z_{i}$s are known, *x* and *y* are not known, and *t* and *z* might or might not be known. Note that $t_{i}$ and *t* are quantified in units of distance (meters), not time (seconds). We square both sides of each equation, obtaining

(8) $$t_{i}^{2}-2t_{i}t+t^{2}=x_{i}^{2}-2x_{i}x+x^{2}+y_{i}^{2}-2y_{i}y+y^{2}+z_{i}^{2}-2z_{i}z+z^{2}\;.$$

In trilateration, *t* is known. In this case, every solution of Equation (7) also solves (8) and vice versa. In multilateration, *t* is an unknown and every solution of Equation (7) corresponds to two solutions of (8): one with $t_{i}-t>0$ and one with $t_{i}-t<0$. The first satisfies Equation (7), but the second does not. We show below how to address this.

Any solution of a set of equations of the form (8) also satisfies differences of equations. This is useful, because in the differences the quadratic terms cancel out:

$$\begin{array}{ccc} & & t_{i}^{2}-2t_{i}t+t^{2}\\ & -\\ & & t_{j}^{2}-2t_{j}t+t^{2}\\ & =\\ & & x_{i}^{2}-2x_{i}x+x^{2}+y_{i}^{2}-2y_{i}y+y^{2}+z_{i}^{2}-2z_{i}z+z^{2}\\ & -\\ & & x_{j}^{2}-2x_{j}x+x^{2}+y_{j}^{2}-2y_{j}y+y^{2}+z_{j}^{2}-2z_{j}z+z^{2}\\ & \mapsto \\ & & \left(t_{i}^{2}-t_{j}^{2}\right)-2\left(t_{i}-t_{j}\right)t\\ & & =\left(x_{i}^{2}-x_{j}^{2}\right)-2\left(x_{i}-x_{j}\right)x\\ & & +\left(y_{i}^{2}-y_{j}^{2}\right)-2\left(y_{i}-y_{j}\right)y\\ & & +\left(z_{i}^{2}-z_{j}^{2}\right)-2\left(z_{i}-z_{j}\right)z\;. \end{array}$$

We assume that if *t* and *z* are known (trilateration), we have two original equations, that if *t* or *z* are known but not both (trilateration in three dimensions of multilateration in two), we have three original equations, and that if none are known (multilateration in three dimensions), we have four equations. If we think of each of the *m* original or squared equations as a vertex in a graph, we can form at most m−1 linearly independent difference equation. The resulting equations will be structurally independent only if the edges of the graph that correspond to difference equations forms a spanning subgraph with no cycles. For simplicity, we can simply subtract equation i+1 from equation *i*.

We now have m−1 equations in *m* unknowns. We denote this linear system by Au=b. If both *t* and *z* are not known, we have

$$\begin{array}{ccc} A_{i:*} & = & \left[\begin{array}{cccc} 2(x_{i}-x_{j}) & 2(y_{i}-y_{j}) & 2(z_{i}-z_{j}) & -2(t_{i}-t_{j}) \end{array}\right]\\ b_{i} & = & -\left(t_{i}^{2}-t_{j}^{2}\right)+\left(x_{i}^{2}-x_{j}^{2}\right)+\left(y_{i}^{2}-y_{j}^{2}\right)+\left(z_{i}^{2}-z_{j}^{2}\right)\;. \end{array}$$

If *t* and/or *z* are known, the corresponding column of *A* multiplied by that value is subtracted from *b* and dropped from *A*.

Assuming *A* has full rank, the solutions *u* have the form

(9) $$u=U\Sigma^{-1}V^{T}b+\alpha v=w+\alpha v\;.$$

where $A^{T}=U\Sigma V^{{}^{T}}$ is the thin SVD of *A* and *v* is the null vector of *A*. We substitute this expression for $u=\left[\begin{array}{cccc} x & y & z & t \end{array}\right]$ (or with fewer elements for problems with only two or three unknowns) in Equation (8) to obtain a quadratic equation in one unknown, α. That is, we substitute $x\mapsto w_{1}+\alpha v_{1}$, $y\mapsto w_{2}+\alpha v_{2}$, and so on. This normally yields two solutions for α, which we substitute in Equation (9) to obtain two solution vectors to the set of squared Equations (8).

The structure of the quadratic equation is as follows. Consider the term ${\left(x_{i}-x\right)}^{2}$ under the substitution $x\mapsto w_{1}+\alpha v_{1}$,

$$\begin{array}{ccc} {\left(x_{i}-x\right)}^{2} & = & {\left(x_{i}-w_{1}-\alpha v_{1}\right)}^{2}\\ & = & x_{i}^{2}+w_{1}^{2}-2x_{i}w_{1}\\ & & +\left(-2x_{i}v_{1}+2w_{1}v_{1}\right)\alpha \\ & & +v_{1}^{2}\alpha^{2}\;. \end{array}$$

The other terms have the same structure.

If all of these solutions have $t_{i}-t\geq 0$, we are finished. If all the $t_{i}$s are identical and $t_{i}-t<0$, we need to negate the differences,

$$\begin{array}{ccc} t_{i}-t_{\mathrm{fixed}} & = & -\left(t_{i}-t\right)\\ t_{\mathrm{fixed}} & = & t_{i}+\left(t_{i}-t\right)\\ t_{\mathrm{fixed}} & = & 2t_{i}-t\;. \end{array}$$

If the $t_{i}$s are not all the same and some $t_{i}-t<0$, the original equations are inconsistent even though the squared ones are.

## 3. Results

**Figure 3:**
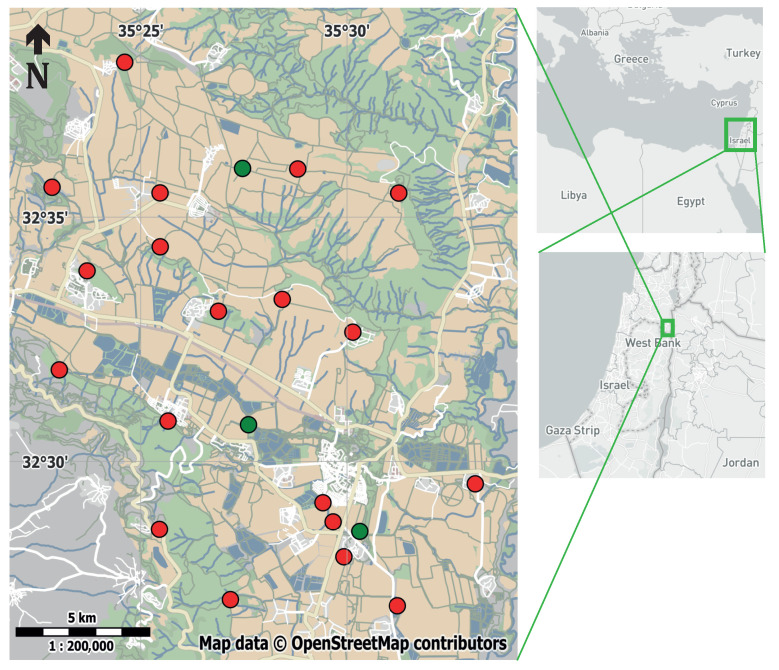
The layout of the ATLAS system in the Harod valley in Israel. Base stations locations are marked by red circles and beacon locations by green circles.

We now present real-world data that show the effectiveness of the new algorithms. The data were generated by an ATLAS system installed in the Harod valley in Israel. The location of base stations and beacons is shown in Figure 3. We use data collected in two separate efforts. One data set is a large study of the movement of wild barn owls (*Tyto alba*) [18] and the other is a data set collected using a transmitter mounted on a car, intended to characterize the accuracy of ATLAS and its location estimation algorithms.

We compare the new algorithms to a baseline version from November 2021. Localizations from the baseline versions were computed in 2D using the simplified (differenced) single-beacon transmission formulation.

Unless otherwise noted, we run the new algorithms with the following parameters: the initial guess is selected using the consensus method, outliers were rejected based on an unweighted residual threshold of 100 m (the weighted residual was subject to a small limit, so most of the time the unweighted threshold classified outliers). The standard deviation of the evolution constraints of the Kalman filter was set to $\min(0.1\Delta_{t},750)$, and the distance parameter of DBSCAN, called epsilon, was set to 50 m.

We label some of the data with the number of base stations (NBS) that were used to produce a localization, and by the standard deviation of the localization in meters (std), which we define to be the square root of the largest eigenvalue of the 2D covariance matrix; this is the standard deviation of the localization along the direction of maximal uncertainty.

### 3.1. Easy Cases

**Figure 4:**
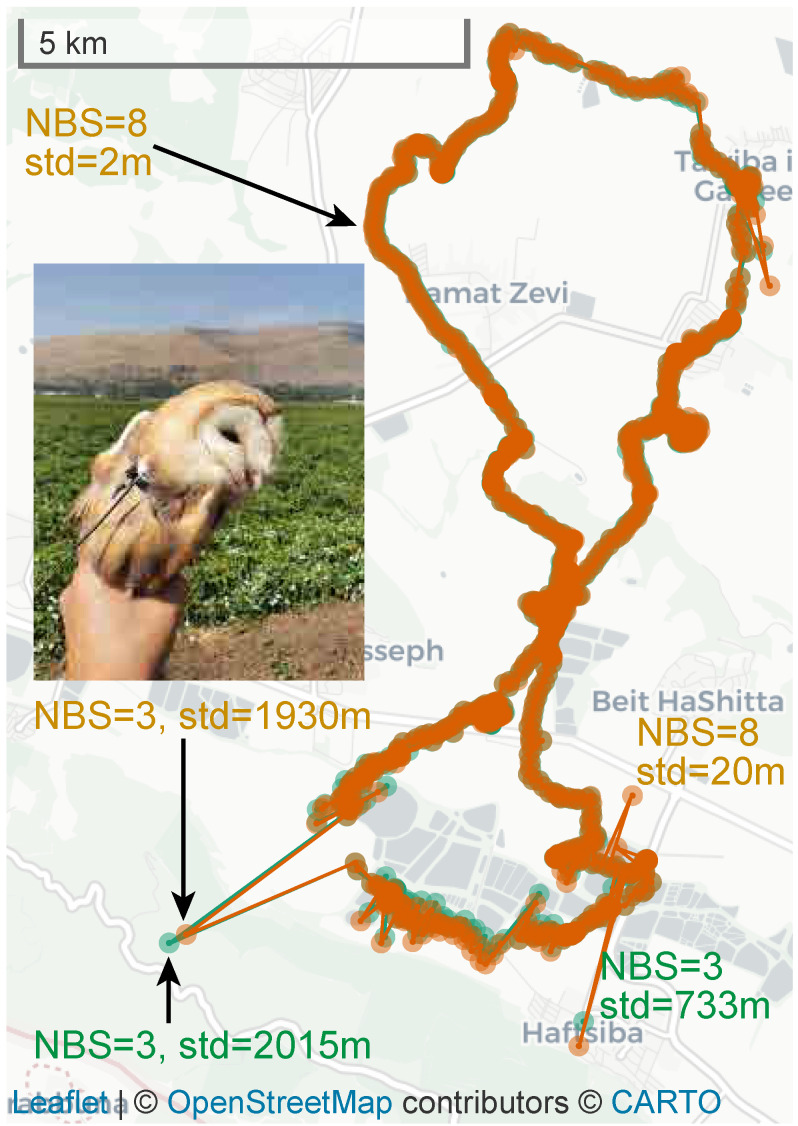
Localizations of one barn owl over one night. Orange dots represents localizations by the new robust algorithm, and green dots, mostly obscured by the orange dots, represents localizations by the baseline algorithm. The tracks show that in easy cases the baseline algorithm performs well and they show that the robust algorithm mostly eliminates outlier localizations derived from more than three detections. Outliers derived from three detections are not removed by the robust algorithm.

In many cases, the baseline algorithm performs well. In such cases, the behavior of the baseline algorithm and the new algorithms is virtually indistinguishable. Figure 4 shows such a case. The two tracks overlap each other. Both suffer from a few outliers, caused by arrival time measurements that are very noisy. The covariance matrices of such localizations indicate clearly that they are inaccurate, as shown in the figure. Most of the tracks produced by ATLAS are this good.

### 3.2. Outlier Rejection

**Figure 5:**
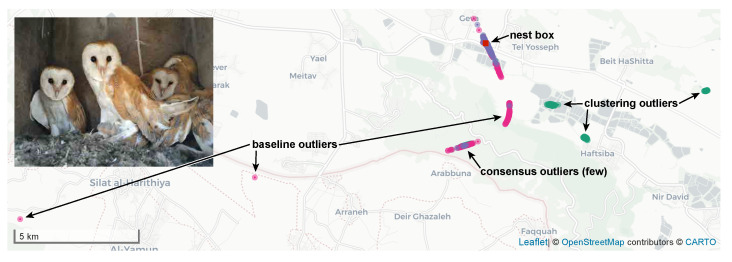
Localizations of a stationary female barn owl that never left its nest box, marked by a red square. Localizations produced by the baseline version are shown in dark pink, those produced by the new robust consensus-based version in purple, and those produced by the new robust clustering-based version in green. Purple dots often obscure pink and green ones underneath. The shape formed by the localizations is typical (see text).

**Figure 6:**
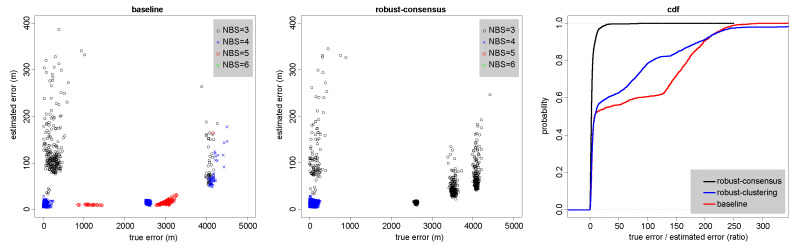
Quantification of the errors in the localizations shown on the map in Figure 5. Errors in the baseline algorithm (**left**), in the new robust consensus-based algorithm (**center**), and the cumulative distribution functions of true-to-estimates error ratios in all three algorithms (**right**). The data is for the localizations shown in Figure 5.

In harder cases, the new algorithms, especially the consensus-based one, are much more effective. Figure 5 and Figure 6 demonstrate the effectiveness of the new consensus-based robust localization algorithm. The figures present data from a set of localizations of a static female barn owl. Owls are cavity breeders where the females as the sole incubator are being fed by their partners and do not leave their nest box [42]. Due to their asynchronous hatching (incubation starts when the first egg is laid), incubation lasts 1–2 months depending on the number of eggs (typical 2–11 [43]). Nest survey confirmed this female was incubating at least three eggs, and accordingly we assumed conservatively that incubation lasted at least 32 days (28-day incubation period and an egg laid every 2 days).

Figure 5 shows that the localizations produced by the baseline version are scattered along a hyperbola, with many gross outliers. This tends to happen when a transmitter is detected by only three receivers, two with good SNR, thereby localizing the transmitter to a hyperbola, and a third with poor SNR, which spreads the localizations along the hyperbola. The localizations produced by the consensus-based robust algorithm are also scattered along the same hyperbola, but much less so. The clustering-based robust algorithm has many more outliers than the consensus-based ones, including at locations that are never generated by the baseline algorithm (to the east).

The leftmost scatter plot in Figure 6 shows that the baseline version produces numerous localizations that are 1–4 km away from the actual location of the owl, with the error in most of them being grossly underestimated. This happens even when the localizations use four or five base stations.

On the other hand, the data for the consensus-based robust version, shown in the center scatter plot, indicates that it produces few outliers, and that virtually all of them have an NBS value of three, making them easy to identify later. The algorithm was able to classify most of the erroneous time-of-arrival measurements as outliers and to discard them (this is why there are no estimates with NBS values of five or six).

The CDFs in the rightmost graph show that the fraction of localizations with approximate standard deviations that are significantly smaller than the true errors is small in the robust consensus algorithm, but large in the two other algorithms.

We stress that difficult cases like this occur mostly under well-understood conditions, many of which are of notable importance for ecological research. In some of these cases, the difficulties arise because the tracked owl reaches the edge of the coverage area. Accurate tracking is much needed in such cases to estimate the size of the home range and the variation in foraging bouts in space and time. Furthermore, female owls commonly remain within the nest box for many days during incubation and early nestling rearing, being fed by their partners [42]. Therefore, studies of breeding success and division of labor (or sex roles) among parents require accurate records also during such stationary periods [42,44,45]. Hence, system performance may also be important in these cases to distinguish false movements outside the nest from real ones.

### 3.3. Tags Close to Base Stations

**Figure 7:**
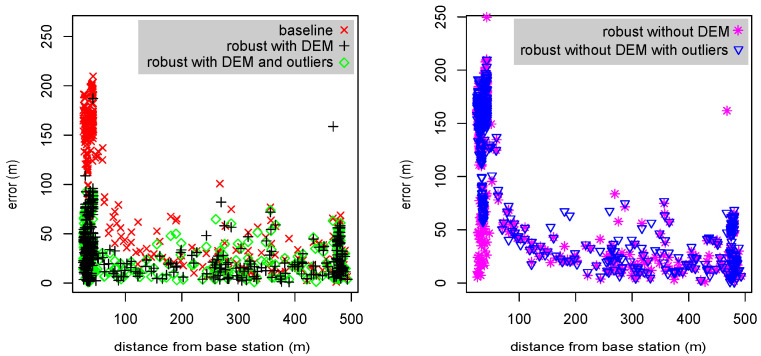
Localization errors as a function of the distance from the base station closest to the tag. The data are from two tags mounted on a car that also carried a GPS device whose localizations are used as ground truth for these graphs. Errors are shown for five different variants of the localization algorithm.

Figure 7 demonstrates the effect of two new algorithmic mechanisms on the localization errors, especially when tags are close to base station. The graphs show that the baseline version generates errors of 100–200 m when tags are close to the base station. This is caused by the height assumption. The new robust version also generates large errors, 50–200 m, in such cases, but only if outliers are not rejected from the final optimization (we implemented an option to include outliers specifically to generate this graph). When a DEM is used, the errors near a base stations are often much smaller. Also, when outliers are rejected, errors near a base station are much smaller; this is caused by the time-of-arrival measurement at the base station closest to the tag being rejected as an outlier when it is inconsistent with the best hypothesis due to the incorrect assumed altitude. This is acceptable, but not as good as using a DEM, because the algorithm is rejecting a measurement that typically has good SNR.

### 3.4. Improvements in Error Estimation

**Figure 8:**
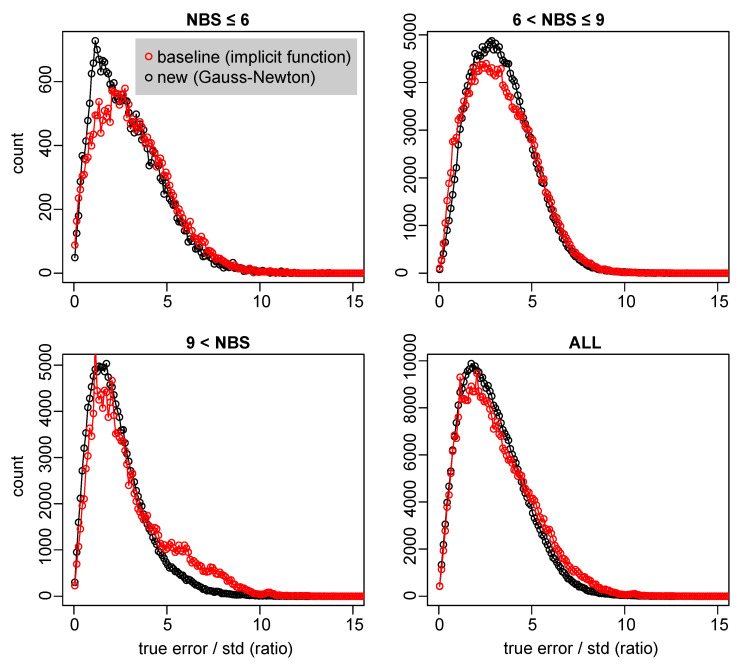
The ratio between the actual localization error and the estimated standard deviation of the error. Reliable error estimates are characterized by a concentration around one. A heavy tail indicates that there are many underestimates of the error. The data is from stationary tags over a period of about 2 weeks. The data is split by the number of base stations (NBS) that produced each localization.

The new Gauss–Newton-based technique for estimating the covariance of localizations produces fewer underestimates of the variance. The data in Figure 6 and Figure 8 show that the new algorithm produces more accurate estimates and fewer underestimates. The data in Figure 8 are from stationary tags that were relatively easy to localize. A larger data set of mobile tags (mounted on a car) from the edges of the system showed a smaller discrepancy between the two algorithms, and slightly more significant underestimate (ratios of 10–20 between the actual error and the standard deviation).

### 3.5. Performance

Localization algorithms designed for high-throughput systems must be efficient, both to cope with real-time data and to be able to recompute the massive quantities of stored historical data. Table 1 shows the running time (on a laptop with an Intel i7-8565U processor running at 2.0 GHz) of several algorithms and combinations of features when they are used to localize one beacon using detections from two other beacons. This is close to the worst-case performance of the algorithms because the beacons are detected by many base stations, often more than 10, due to high placement and high-gain antennas. We do not show data for single-beacon-transmission localization with the legacy algorithm because this algorithm almost always reverts to the use of the multiple-beacons variance when used on beacons.

The data show that the new consensus-based algorithm is faster than the legacy algorithm; in multiple-beacon settings, it runs in about 10 ms per localization without snapping to a DEM and in about 13 ms with snapping, compared to 14 and 17 ms for the legacy algorithm. In the single-beacon transmission setting, the consensus-based algorithm is much faster, taking about 1 ms without snapping to a DEM and about 1.7 ms with snapping.

This is good performance. From the spectrum utilization standpoint and from the base station performance standpoint the throughput of a large single0 channel ATLAS system can reach about 100 localizations per second. A localization server using the single-beacon consensus-based algorithm can easily cope with this load, and even the multiple-beacons setting is manageable.

The clustering algorithm is about 4–5 times slower. This is mostly due to the performance of the implementation of DBSCAN that we use. We have not yet explored other implementations or alternative clustering algorithms.

## 4. Discussion

The new algorithms that we have presented are novel, effective, fast, and they resolve or significantly mitigate actual failures of the baseline algorithm.

Of the two methods to generate a good initial guess, our results suggest that the consensus method is both more reliable and much faster than the clustering method. However, we have spent significant efforts on tuning the consensus method and less efforts on tuning the clustering method, so it too might be made competitive. Also, our results do not rule out the possibility that the clustering method is more effective than the consensus one in some specific cases, such as localizations from observations by many receivers.

Our consensus and outlier classification rules are applied to differences of two constraints, each based on one raw observation. Therefore, if one observation is an outlier, we discard both. A method to classify individual constraints might discard fewer observations.

The way we use a DEM, by computing a 2D estimate, looking up its elevation in the DEM, and then computing one more 2D estimate with the altitude snapped to the DEM’s elevation is simple and works well in practice but is not optimal. A more reliable approach would be to go through iterations of 2D localization and snapping until reaching a fixed point. Another approach, which focuses on the model rather than on the algorithm, is to use the DEM to define a differentiable function elevation(x,y) and to add the constraint z=elevation(x,y) to the least-squares problem.

The new algorithms are fast enough to not become a bottleneck in most ATLAS servers, but their performance can probably be significantly enhanced, both by tuning algorithmic parameters, and by replacing the library implementations of key subroutines by faster ones (e.g., replacing the SVD from the Apache Commons Math library by the SVD from LAPACK, or by using a GPU, etc.).

## Figures and Tables

**Table 1:** CPU Time (average number of milliseconds) per localization using several algorithms and combinations of optional features. The data are derived from runs on one hour of data from three beacons. Each entry represents the median of three runs. We do not show the performance of the single-beacon baseline algorithm because it usually reverts automatically to use multiple beacons when invoked on beacons (because they are received by many base stations).

	Single	Multiple	DEM Single	DEM Multi
consensus	1.0	10	1.7	13
clustering	37	51	35	51
legacy	-	14	-	17
